# Teaching Youth to Resist Abuse: Evaluation of a Strengths-Based Child Maltreatment Curriculum for High School Students

**DOI:** 10.1007/s40653-020-00304-2

**Published:** 2020-03-10

**Authors:** Marisol J. Diaz, Wendy Wolfersteig, Diane Moreland, Grant Yoder, Patricia Dustman, Mary L. Harthun

**Affiliations:** Southwest Interdisciplinary Research Center, School of Social Work, Watts College of Public Service and Community Solutions, Arizona State University, Downtown Phoenix Campus, 201 N. Central Ave., 33rd floor, Phoenix, AZ 85004 USA

**Keywords:** Adolescents, Child maltreatment, Curriculum, Evaluation, Prevention

## Abstract

Child maltreatment (CM) is a serious and prevalent public health problem in the United States (U.S.) yet programming to combat the issue often overlooks high school aged youth (those aged 14–17). In 2017, over 90,000 youth in the U.S. experienced CM during their high school years (U.S. Department of Health and Human Services 2019). This manuscript will highlight the importance of prevention programs for youth affected by child maltreatment and report the findings of a pilot study that examined the effectiveness of the Childhelp Speak Up Be Safe Prevention Education Curriculum among high school students. The purpose of the pilot study was to determine if the revised and expanded curriculum for grades 9–12 was feasible and to examine the validity of the new survey items, including the RESIST strategy questions. The pilot study utilized a two-phase non-probability convenience sample to evaluate high school student gains in knowledge of safety related resistance strategies. High school students (*N* = 269) attending one urban charter public high school (grades 9–12) in the Southwest who completed pre- and post-survey RESIST strategy items participated in the pilot. The results indicated that students receiving the Childhelp Speak Up Be Safe Prevention Education Curriculum increased their identification and knowledge of safety related resistance strategies.

## Background

Child maltreatment (CM), a serious and prevalent public health problem in the United States (U.S.), is responsible for substantial mortality (Fang et al. 2012). In 2017, there were 674,000 U.S. victims of child abuse and neglect with an estimated 1720 deaths, which is one of the worst rates among industrialized nations (U.S. Department of Health and Human Services [DHHS] 2019). The U.S. has made progress in preventing child abuse and neglect (Finkelhor and Jones 2006, 2012); however, child abuse rates have remained steady with some increases (Finkelhor et al. 2018a). Each year, hundreds of thousands of cases of CM are reported to Child Protective Services (CPS), and it is CPS’ responsibility to decide what, if any, response should be made to ensure the child’s safety (Fluke et al. 2008). During federal fiscal year 2017, CPS agencies received an estimated 4.1 million referrals involving approximately 7.5 million children (DHHS 2019). The increase in referrals has resulted in substantial increases in CPS investigations. The national estimate of children who received a CPS response rose from approximately 3,184,000 in 2013 to 3,501,000 in 2017, representing a 10% increase (DHHS 2019).

Age is an important risk factor for exposure to CM. In 2017, the national rate of victimization for high school was 9.1 per 1000 youth, equating to over 90,000 youth in the U.S. who experience CM during their high school years (DHHS 2019). These numbers correspond with national statistics that show three-quarters (74.9%) of victims are neglected, 18.3% are physically abused, and 8.6% are sexually abused (DHHS 2019). Additionally, there is strong evidence that young adolescence represents the riskiest period for perpetrating sexual harm against younger children (Letourneau et al. 2017). Therefore, school-based prevention programs that aim to educate adolescent youth about the different types of abuses may have the promise to prevent a sizable portion of CM in an efficient manner (Letourneau et al. 2017).

In the U.S., the effects of CM are far-reaching and long lasting. Peterson et al. (2018) estimate the cost of CM in the U.S. to be approximately $428 billion dollars annually. In regard to academic achievement, youth who experience CM are rated lower by teachers, score lower on standardized tests, obtain lower grades, are suspended more frequently and are more likely to be held back (Slade and Wissow 2007). Fourteen percent of all men in prison and 36% of women in prison in the U.S. were abused as children, about twice the frequency seen in the general population (Harlow 1999). Furthermore, CM has a tremendous effect on the health and wellbeing of those youth who are impacted. CM is associated with higher mortality rates, obesity, HIV, mental health issues, suicide and criminal behavior (Wildeman et al. 2014). Given the rates of CM for high school youth, and the societal costs of these issues, there is a need for programming targeted to this age group. While there are a few existing programs for high school aged youth, such as Play it Safe!®, most of existing research has centered on elementary aged youth (Blakey and Thigpen 2015).

### Prevention Programming

Given the mounting evidence about the prevalence of CM, school-based child safety programs are an increasingly popular method to address this issue (Finkelhor et al. 2018b). Thus, CM prevention programs are not a new concept, but there are some methodological and implementation issues associated with many of the programs. Many of the CM prevention programs tend to be single-harm focused, for example, focusing on only one type of abuse -usually sexual abuse (Dale et al. 2016). Multiple studies examined the impact of child sexual abuse prevention programs. Several of these studies are meta-analyses examining the effects of multiple programs. These analyses reported a wide array of findings, but generally found positive effects associated with participation in prevention programming. One study conducted by Davis and Gidycz (2000), examining 27 sexual abuse prevention programs, found an average effect size of 1.07; this corresponds to a large effect size (Cohen 1992). Meta-analyses by Heidotting et al. (1994) and by Topping and Barron (2009) identified similar positive effects, albeit with smaller effect sizes. Heidotting et al. (1994) examined the effects of 18 different programs, and calculated an average effect size of .57, and Topping and Barron (2009) analyzed 22 studies with an average effect size of .61, both corresponding to a moderate effect size (Cohen 1992). Common across all of these programs, importantly, was the lack of programming specific to high school aged youth. Only one study in the meta-analysis included older youth, and it was specifically for those in college, with ages ranging from 16 to 28 (Gibson and Leitenberg 2000). The lack of evidence-informed child abuse programming for high school youth represents a significant gap in existing research.

There are numerous programs designed to address CM, many of which have promising results; likewise, there are some common limitations (Walsh et al. 2015). As previously discussed, many CM prevention programs are single-harm focused, and may overlook other forms of CM (White et al. 2018). The single-harm focus could represent a missed opportunity for improving child wellbeing and reducing CM. Given that single-harm focused programs often emphasize similar skills (e.g., identifying safe situations and developing interpersonal skills), integrating multiple prevention programs may be more efficient (White et al. 2018). There is a tremendous need for programming related to neglect, as there are no manualized evidence-based school programs for the prevention of psychological effects of physical neglect (Brassard and Fiorvanti 2015). Programs which focus on multiple forms of maltreatment could be an important part of ensuring youth have the skills to protect themselves from maltreatment. Presently, there is little research being conducted to ensure the safety of high school aged youth exposed to or experiencing CM. It is therefore critical to develop effective programming for this traditionally overlooked population while also ensuring rigorous evaluation of any such program.

As Topping and Barron (2009) indicated, many of the studies on CM focus primarily on White populations. A further criticism leveled at these programs is a general lack of rigor in the evaluation procedure. It is argued that many of these programs are delivered universally without sufficient evidence to justify their continued implementation (Dale et al. 2016; Pulido et al. 2015). As such, large-scale efficacy trials are necessary to make informed decisions about the effective implementation of these types of programs (Peterson et al. 2018). More research is necessary to understand how best to serve high school aged youth with CM prevention programming. Currently, there is a major gap in the CM literature for this age group and the Childhelp Speak Up Be Safe Curriculum is designed to help address this gap. The pilot findings represent the first step in a rigorous evaluation of this CM program designed to serve high school aged youth. The Childhelp Speak Up Be Safe restructured curriculum and systematic evaluation were designed to address many of the methodological and evaluative issues identified in previous research.

### Evolution of Childhelp Speak Up be Safe Prevention Education Curriculum

In 2011, Childhelp, the nation’s oldest and largest nonprofit advocating for abused and neglected children, was awarded a U.S. Department of Education Grant to revamp the Good Touch, Bad Touch prevention education program to address up-to-the-minute concerns such as Internet predators and bullying. A childhood expert and research team completed the work, and the curriculum (for grades 1–6) became Childhelp Speak Up Be Safe, the first version. Florida became the first state to mandate prevention education in every school (over 400 school districts) and chose Childhelp Speak Up Be Safe as its official program. Childhelp then engaged another university to expand the program to pre-K through 12th grades. The Childhelp Director of Curriculum described this second version of the curriculum as incohesive and in 2014–2015, Childhelp initiated a partnership with the Southwest Interdisciplinary Research Center (SIRC) to complete evidence-informed revisions for all grades. Over a nine-month period, the SIRC team developed a third version of the curriculum with the understanding that an implementation feasibility pilot would be essential prior to conducting a randomized control trial (RCT) of a final version.

In developing the third version, the SIRC research team determined that the application of ecology as a holistic theoretical approach was fundamental, as children and adolescents do not exist in isolation. Youth are entrenched within a larger social structure interconnected with other social institutions and dynamics. Thus, the curriculum was re-designed using the social-ecological systems theory as a framework to empower students with the skills they needed to play a significant personal role in the prevention or interruption of abuse, neglect, bullying, and promotion of internet safety in multiple environments. Bronfenbrenner’s social-ecological model conceptualizes the social ecology of the individual as a set of five interacting environmental systems that are linked and influence child development (Bronfenbrenner 1979, 1986). The ecological systems theory targets environmental risk and protective factors at different levels as essential effects influencing the impact of prevention messages (Bronfenbrenner 1979). Following Bronfenbrenner’s ecological theory, Belsky (1980) laid out an ecological model of the etiology of CM. Similarly, this model explains different factors that contribute to the risk for CM, mostly a combination of individual, relational, community and societal factors that can be associated with all different types of abuse. Building on protective factors within children’s environments and integrating those with a primary prevention approach served as the framework for SIRC researchers in re-designing the curriculum.

**Fig. 1:**
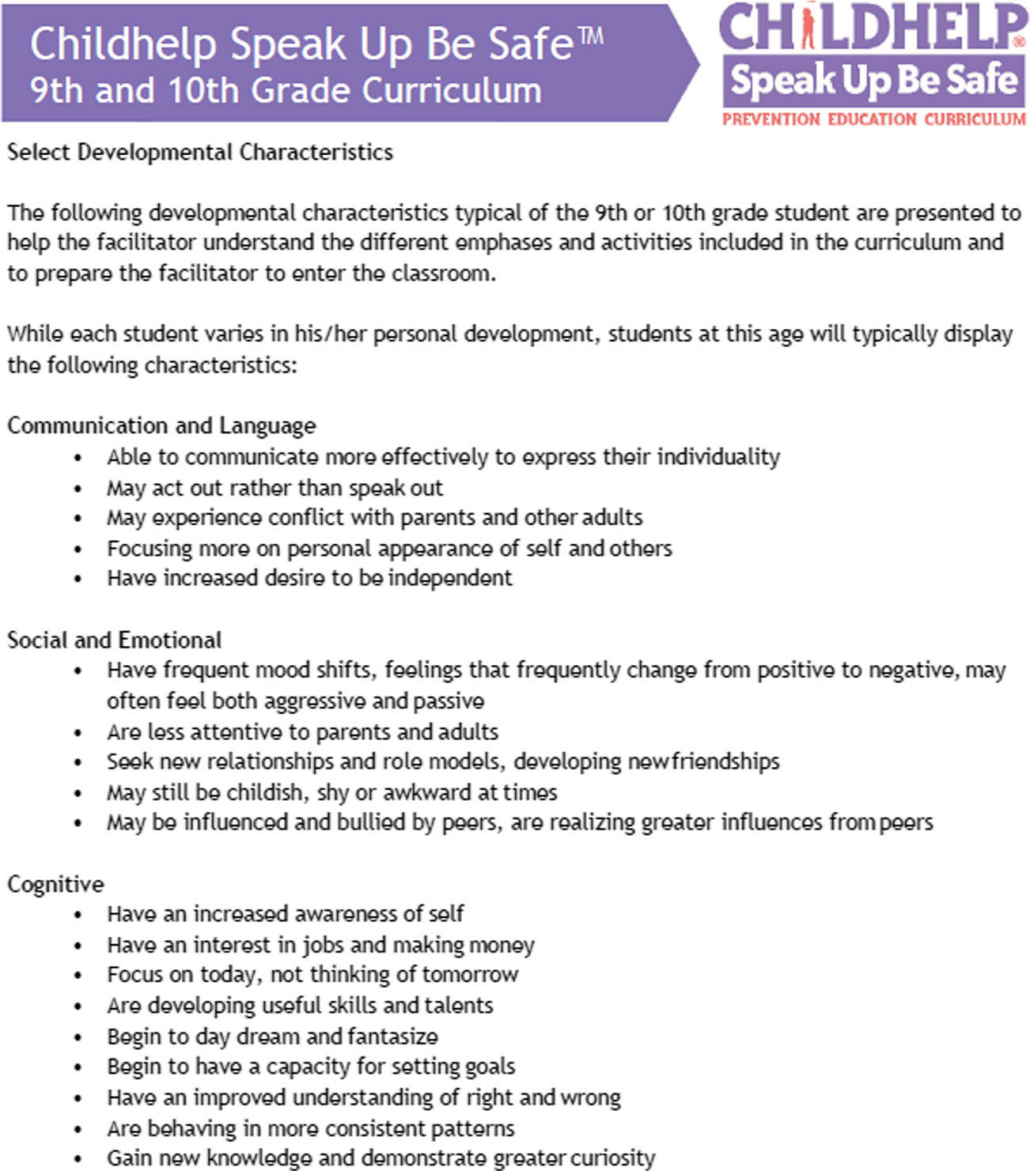
Childhelp Speak Up Be Safe 9th and 10th Grade Curriculum

Each grade lesson was designed with child development, learning styles, and social psychology in mind. For each lesson, there are PowerPoint slides, big ideas, key terms, examples, and activities/exercises. Students learn age-appropriate definitions and participate in class activities to recognize what they need to stay safe and healthy. The curriculum connects the responsibility of adults and community members to keep children safe. A variety of methods are used to present the material, including direct instruction, discussion segments with questions and answers, and scenarios with guided small group conversations. To help facilitators understand how the “big ideas” and in-class activities of the lesson are appropriate for the ages of the children, the SIRC research team added and defined developmental characteristics of students at each grade level. Figure 1 is an example and illustrates how the developmental characteristics were included in the lesson plan for the facilitators for grade 9 and 10 (see below).

Another adaptation included identifying the “big ideas” for each lesson to ensure that age appropriate messages were taught, and the content was aligned from grade to grade. The team reframed the 5 Safety Rules taught in lower grades to the 5 Safety Principles for high school students and expanded the concepts of each principle. For example, in upper grades, the discussion about Safety Rule #1, It’s My Body, becomes a Safety Principle, “I decide what to do with my body.” Students learn they have autonomy over their bodies; they make decisions, free from coercion, about who gets to touch them. They learn that no one has the right to force a hug on them; they consent to who hugs, and who does not. As the age and grade of the students increase, the emphasis of responsibility shifts from children depending on a trusted adult to help them understand the importance of neglect and abuse, to adolescents taking a more active role in identifying what they need personally to be healthy, safe, and cared for while also identifying people who can help them.

### Pilot Study

In 2016, SIRC conducted the initial external evaluation of the third version of the Speak Up Be Safe Curriculum through a pilot study. The overall goal of the pilot was to test the effectiveness of the revised curriculum in all grades and use feedback from various data sets to adjust the curriculum, implementation processes, online training, surveys, and survey administration prior to implementing a full-scale RCT. Although the Speak Up Be Safe curriculum is designed for pre-kindergarten to grade 12, the current discussion focuses only on grades 9–12 and speaks to the implementation and outcomes for this target population. An evaluation was done of all survey items however; the RESIST strategies taught within the high school Speak Up Be Safe curriculum are one of its key concepts and the focus of this paper. The high school students received the curriculum in the two-lesson format with the students completing a pre-survey preceding the first lesson, to obtain baseline knowledge, and a post-survey following both lessons to determine knowledge acquisition. There were six RESIST strategy questions on both the pre- and post-surveys. Each survey was aligned to curriculum key terms and concepts presented and adjusted for reading level based on grade. The surveys were administered online using Qualtrics software to decrease time and resources spent in collecting and cleaning data.

There are two sections within the Speak Up Be Safe curriculum that highlight how to apply resistance behavior in the form of taking action against potential or real abuse, (a) Resistance Strategies and (b) Practicing Safety Principles and Resistance Strategies. These sections detail the importance of adopting the resistance strategies, as well as discussing examples of ways to prevent and stop abuse. A group discussion prompts the students to engage in the content and to generate examples of RESIST strategies for both in-person abuse and cyber abuse. In the practice section, the facilitator asks students to get into groups of four or five and practice recognizing potentially abusive situations and possible solutions. They are six to eight scenarios (six scenarios for grades 9/10 and eight scenarios for grades 11/12) with the intent to encourage students to apply what they learned in the lessons. Each scenario asks four questions of the students,

- Is this abusive behavior? By whom?
- How do the personal safety principles apply?
- What RESIST strategies could be used?
- Is this a healthy or unhealthy relationship? Why?

## Method

Prior to the start of the pilot, counselors completed the online curriculum facilitator training developed by SIRC which included universal modules on child abuse and neglect as well as modules specific to the grade levels they were facilitating. In exchange for their participation, the pilot schools received access to the online curriculum and facilitator training at no charge for two years. Social workers/counselors also participated in a 1.5-h evaluation protocol training, including survey administration processes. The high school pilot and evaluation study utilized a pre-post design with one group. The pre- and post-surveys were developed for the pilot and included items pertaining to resistance strategies from the Childhelp Speak Up Be Safe Curriculum. The revised items were based on key terms and concepts taught within each grade level. Pre- and post-surveys contained the same outcome items while the post-survey also included additional program evaluation items. Data from the pre- and post-surveys were analyzed, and along with focus group data, were used to identify additional changes for the RCT.

## Participants

The high school pilot study utilized a two-phase non-probability convenience sample of students attending one urban charter public high school (grades 9–12) in the southwestern U.S. All four high school grades (9–12) were invited to participate in the pilot. Demographic data including grade, age, gender and ethnicity were collected as part of the pilot. Analysis of the demographic data showed that 9th through 12th grade students (*N* = 269) completed pre- and post-survey RESIST strategy items. There were 74 (27.5%) 9th graders, 74 (27.5%) 10th graders, 68 (25.3%) 11th graders, and 53 (19.7%) 12th graders. Students’ ages ranged from 14 to 19 years of age with a mean age of 16. Over half of the high school students were female (57.2%). Students were asked to select all that apply for the race/ethnicity question. Approximately two-thirds reported an ethnicity of Hispanic (74%). Twelve students or 4.5% preferred not to answer the race/ethnicity question (see Table 1).

**Table 1:** Demographics

Variables	
**Sample size**	269
**Age** (average)	16
**Sex (%)**	
Male	39.4
Female	57.2
**Race/ethnicity (%)**	
Hispanic/Latino	74.0
White	14.9
African American or Black	13.4
American Indian or Alaskan Native	3.3
Asian or Pacific Islander	6.7
Other Ethnicity	4.5
Prefer Not to Answer	4.5

## Measures

The pre- and post-surveys were developed specifically for this curriculum to measure the concepts of safety knowledge, safety rules, resistance strategies, and safety scenarios taught. While some standardized measures exist for these concepts, the study team did not find measures that were specific to how the learning objectives and key terms are taught within each grade level. Thus, the team developed measures specific to the strategies as operationalized, with the pilot test important to confirming measurement validity and reliability. A readability analysis was conducted for each item, using the Flesch-Kinkaid Grade Level Test, to assess and ensure age appropriateness. This manuscript highlights results of the resistance strategy items. In the curriculum, students are taught that RESIST stands for Run, Escape, Scream, Ignore, Stay Away and Tell. On both the pre- and post-surveys, students were presented the first letter of each RESIST strategy and asked to select the word that corresponds with the first letter from a choice of three words. Each item had several strategy choices and the correct RESIST strategy as taught in the curriculum is shown in bold (see Table 2).

**Table 2:** RESIST Items and Responses with Correct Response in Bold

What does R mean?	Reach	Repeat	**Run**
What does E mean?	Enter	**Escape**	Eat
What does S mean?	Sit	**Scream**	Stand
What does I mean?	Imitate	Invite	**Ignore**
What does S mean?	**Stay Away**	Stay in Place	Stand
What does T mean?	Turn	**Tell**	Text

### Procedures

#### Institutional Review Board

The pilot school district had an existing Arizona State University (ASU) Human Subjects protocol in place that covered the approval for implementation of the pilot curriculum and surveys. Therefore, all protocols and instruments for data collection were reviewed and approved by the Social Behavioral Institutional Review Board (IRB) at ASU as modifications to the existing protocol, and team members were added as well. Because of the overall protocol, parental permission was already granted; however, parents did receive the name and contact information of the principal investigator in case of any questions or concerns, and the surveys were available in school offices for parent review.

#### Data Collection

Surveys were administered by the school facilitators in April and May 2017 with seven weeks between pre- and post-survey completion; students completed the pre-survey before receiving the first lesson and the post-survey following the second lesson. Both surveys were administered online using Qualtrics software. An online survey link was sent to the facilitator prior to the administration of each type of survey. The facilitator shared the link with the high school students. Students were each assigned a unique identification number that was entered by the student while completing each survey in order to link the pre- and post-survey data. Students averaged 15 min to complete the pre-survey and 24 min to complete the post-survey. Data were downloaded from Qualtrics and imported into SPSS for analysis.

## Results

### Data Analysis

As a part of each survey, students were asked a series of questions related to the RESIST strategies taught. Responses to the RESIST questions were dichotomized to reflect correct (1) and incorrect (0) responses. An initial factor analysis using principal component analysis was conducted on the six-resistance strategy (RESIST) items. The analysis yielded two factors: one with five-items and one with a single item. Two possible reasons can help explain why the T was not included. The first is that participants may have guessed the correct answer of Tell, as the response could be intuitively linked to the title of the curriculum, Speak Up. The second feasible explanation is that participants may have had exposure to some prevention concepts in earlier lessons in which *tell* is a common safety strategy. A second factor analysis using principal component analysis was then conducted with the five-items only. The five-items were Run, Escape, Scream, Ignore and Stay Away. The analysis yielded a new Resistance scale and explained 52.503% of the variance (Table 3). Internal consistency was examined for the Resistance scale using Cronbach’s alpha. The alpha was acceptable at .76 (Tavakol and Dennick 2011) .

**Table 3:** Factor Analysis Table for Run, Escape, Scream, Ignore and Stay Away Items

	Component 1
R =	.733
E =	.711
S =	.823
I =	.675
S =	.670
Eigen Value	2.625 s
% of Total Variance	52.503

Next, a cumulative composite score was calculated by adding the number of correct answers in the set of five questions for each student. Possible correct composite scores ranged from 0 to 5. Then a paired-samples t-test of the overall composite score was conducted and yielded significantly higher results on the post-survey (M = 4.87, SD = .46) than on the pre-survey (M = 4.19, SD = 1.29), t (268) = −8.549, *p* < .001, d = −.52124 (see Table 4). High school students appeared to gain safety related resistance strategy knowledge after participating in the pilot program.

**Table 4:** Results for Paired-samples T-tests of Overall Scores

	N	Mean	SD	t	DF	p	*d*
Pre-score	269	4.19	1.29				−.52124
Post-score		4.87	.46				
Pre –Post score			1.31	−8.549	268	.000	

Individual scores also were examined for pre- and post-survey differences. Pre-survey scores ranged from 71% to 93% correct and post-survey scores ranged from 94% to 100% correct. Students’ scores averaged 84% on the pre-survey and 97% on the post-survey (see Table 5).

**Table 5:** Resistance Knowledge Scores

*N* = 269	Pre-Survey (% correct)	Post-Survey (% correct)
R = (run)	.71	.94
E = (escape)	.93	1.00
S = (scream)	.82	.99
I = (ignore)	.83	.97
S = (stay away)	.91	.99

## Discussion

### Value of this Evidence

The evaluation of the Childhelp Speak up Be Safe curriculum pilot study adds new evidence to the literature about CM prevention programming. Specifically, through the examination of prevention programming for high school aged students, a population neglected in child abuse research is now being addressed. As CM continues to magnify as a public health issue, it is critical to develop efficacious programming for high school youth. Although child abuse prevention programming is not uncommon, with nearly two-thirds of youth receiving some type of instruction (Pulido et al. 2015), these existing programs are often single harm focused, tailored to younger children, or not evidence-based. Moreover, prevention programming sample populations are predominantly white youth, although minority youth are disproportionately found in the child welfare system (Child Welfare Information Gateway 2016).

At the completion of the intervention, SIRC staff conducted focus groups with the schools’ counselors to gather feedback on the curriculum. The focus groups had the school counselors identify their experiences in delivering the curriculum. In regard to the specific question, what were the most important messages students took from the lessons you facilitated, the focus group participants reported that the group discussion on resisting abuse was “a powerful activity because making the examples personal really helped the kids.” Additionally, the counselors highlighted how the RESIST strategies worked with the high school students, “they were able to identify ways to respond to different scenarios” and “students were more familiar with examples of abuse including examples in which they could be possibly sexually abusing others via cyberbullying.” The counselors felt it was eye opening for students to know more about internet safety and more confidently be able to identify what private information to share as well as what not to share. Currently, there is an imperative need for programming to teach high school students the skills and strategies essential to resist the different forms of abuse and neglect and to participate in their own personal safety and protection behaviors. The Childhelp Speak Up Be Safe Curriculum is unique in that it is designed to teach knowledge and resistance skills related to multiple forms of child abuse and neglect, and to be developmentally appropriate for high school aged youth, including youth of color. These pilot study resistance strategy results suggest that the Childhelp Speak Up Be Safe Curriculum may be successful for a universal audience of youth in grades 9–12.

#### Degree of Rigor

One common criticism of CM interventions is the lack of sufficient rigor to warrant widespread dissemination (Dale et al. 2016). Taken on their own, these pilot results only begin to demonstrate the evidence needed for widespread dissemination of the program. Importantly, these positive initial findings indicate that additional testing is warranted for examining overall program effectiveness and efficacy. The high school students who participated in the program experienced significant improvements in their understanding of the safety strategies taught during the program as measured by the Resistance Scale. The change in resistance understanding responded to an effect size of d = −.52, an acceptable effect size (Cohen 1992). In addition to finding statistically significant differences between the youth who participated in the Childhelp Speak Up Be Safe Curriculum and those who did not, the calculated effect size highlights the practical significance of this effect. The program aimed to increase student knowledge about child abuse resistance strategies. Students who participated in the program did appear to gain resistance strategy knowledge.

This effect size also aligns closely with previously implemented prevention programming. In the meta-analysis of sexual abuse prevention programming conducted by Davis and Gidycz (2000), the average effect size for programming with two or three sessions was d = .63. Further, the meta-analyses Heidotting et al. (1994) and Topping and Barron (2009) calculated average effect sizes of .57 and .61 respectively, both of which correspond to a moderate effect size (Cohen 1992). Any minor differences may be partially explained by the programmatic focus on younger children in the other studies. While it may be expected that older children would learn most from prevention programs, the smaller effect sizes may be explained by a ceiling effect experienced by youth who may have already been exposed to some prevention concepts related to substance abuse prevention or other health-related topics (Davis and Gidycz 2000).

#### Empowerment through Knowledge and Practice

Teaching youth of all ages the skills and strategies needed to recognize and resist abuse is an important step in preventing the pervasive and costly problems of CM. This is especially true for high school aged students who can take an active role in their own protection and personal safety. Although still at risk for experiencing abuse, these students have been overlooked by previous prevention research, as have youth of color. The high school participants taught the Speak Up Be Safe curriculum demonstrated significant improvements in prominent resistance strategies (see Table 5). These strategies provided youth with critical skills to respond appropriately to all four types of abuse, including neglect. Importantly, high school students learned the skills and strategies emphasized in the Childhelp Speak Up Be Safe Curriculum and experienced significant improvements in their comprehension of the RESIST strategies.

### Limitations

Results from this study should be interpreted within the framework of minor limitations. The overall positive pilot findings lend credible evidence to a promising program status of the Childhelp Speak Up Be Safe Curriculum, while a RCT (which is underway) will demonstrate the degree to which it is efficacious and whether or not generalizability as an evidence-based program across a broad spectrum of populations is confirmed. Although this was a simple research design, it is important to point out that this was a pilot study without a control group, thus limiting the ability to attribute certain effects. Moreover, results relied on the self-reported data from high school students who were taught by two different school counselors. While extensive lesson plans and training were provided to the facilitators to help standardize implementation, the researchers were not able to observe the lessons being taught and concede there is always variation in the way individual facilitators deliver the content.

## Conclusions

CM is a serious public health issue for all age groups. A need exists for CM prevention programs to target high school youth as current programs largely focus on elementary children, are single-harm focused, and not evidence-based. To collect evidence, testing the Childhelp Speak Up Be Safe Curriculum began with this pilot study aimed to determine what, if any, specific elements needed to be addressed prior to conducting an RCT across multiple sites. As part of the pilot, the resistance survey items were tested for both validity and reliability. The pilot study findings indicated that participation in the Childhelp Speak Up Be Safe Prevention Education Curriculum increased students’ identification of key safety related resistance strategies. These findings contribute to the literature on the importance and implications of teaching adolescent urban high school youth, a majority of whom were of Hispanic background, how to interrupt and prevent abuse as a way to increase personal safety. Prevention programs like Speak Up Be Safe have the promise to prevent a sizable portion of CM in a cost-effective manner. Future studies on this type of prevention programming for high school students may show results that lead to a reduction in CM, an increase in better health outcomes for youth, and prompt advocates to address the gap in prevention programming for high school youth. Evidence-based programs are becoming a standard requirement for funders and drive policy formation as well as future program development. To meet this rising demand, the implementation of an RCT for Childhelp Speak Up Be Safe Prevention Education Curriculum has begun using a pre-, post-, and follow-up group design with expectations to submit results to a national evidence-based clearinghouse. The RCT, now in progress, is intended to gather evidence of curriculum efficacy at each grade level.

## References

[CR1] Belsky J (1980). Child maltreatment: An ecological integration. American Psychologist.

[CR2] Blakey JM, Thigpen JW (2015). Play it safe!®: A school-based childhood physical and sexual abuse prevention program. Journal of Adolescent and Family Health.

[CR3] Brassard MR, Fiorvanti CM (2015). School-based child abuse prevention programs. Psychology in the Schools.

[CR4] Bronfenbrenner, U. (1979). The ecology of human development. Harvard University Press.

[CR5] Bronfenbrenner U (1986). Ecology of the family as a context for human development: Research perspectives. Developmental Psychology.

[CR6] Child Welfare Information Gateway (2016). Racial disproportionality and disparity in child welfare.

[CR7] Cohen J (1992). A power primer. Psychological Bulletin.

[CR8] Dale R, Shanley DC, Zimmer-Gembeck MJ, Lines K, Pickering K, White C (2016). Empowering and protecting children by enhancing knowledge, skills and well-being: A randomised trial of learn to BE SAFE with Emmy TM. Child Abuse & Neglect.

[CR9] Davis MK, Gidycz CA (2000). Child sexual abuse prevention programs: A meta-analysis. Journal of Clinical Child Psychology.

[CR10] Fang X, Brown DS, Florence CS, Mercy JA (2012). The economic burden of child maltreatment in the United States and implications for prevention. Child Abuse & Neglect.

[CR11] Finkelhor, D., Bright, M. A., Huq, M. S., & Miller, D.M. (2018a). Evaluation of the Monique*. Burr Foundation for Children’s MBF Child Safety Matters Curriculum.* https://www.mbfpreventioneducation.org/wp-content/uploads/2018/07/Research-Study_2018_Report.pdf

[CR12] Finkelhor D, Jones L (2006). Why have child maltreatment and child victimization declined?. Journal of Social Issues.

[CR13] Finkelhor, D., & Jones, L. M. (2012). Have sexual abuse and physical abuse declined since the 1990s? Crimes against Children Research Center. https://scholars.unh.edu/ccrc/61

[CR14] Finkelhor, D., Saito, K., & Jones, L. (2018b). Updated trends in child maltreatment, 2016. Crimes against Children Research Center. University of New Hampshire.

[CR15] Fluke JD, Shusterman GR, Hollinshead DM, Yuan YYT (2008). Longitudinal analysis of repeated child abuse reporting and victimization: Multistate analysis of associated factors. Child Maltreatment.

[CR16] Gibson LE, Leitenberg H (2000). Child sexual abuse prevention programs: Do they decrease the occurrence of child sexual abuse?. Child Abuse & Neglect.

[CR17] Harlow, C. W. (1999). Prior abuse reported by inmates and probationers. U.S. Department of Justice, Office of Justice Programs, Bureau of Justice Statistics.

[CR18] Heidotting, T., Keiffer, S., & Soled, S. (1994). A quantitative synthesis of child sexual abuse prevention programs. American Educational Research Association, New Orleans, LA, April 4-8, 1994. https://eric.ed.gov/?id=ED376217

[CR19] Letourneau EJ, Schaeffer CM, Bradshaw CP, Feder KA (2017). Preventing the onset of child sexual abuse by targeting young adolescents with universal prevention programming. Child Maltreatment.

[CR20] Peterson C, Florence C, Klevens J (2018). The economic burden of child maltreatment in the United States, 2015. Child Abuse & Neglect.

[CR21] Pulido ML, Dauber S, Tully BA, Hamilton P, Smith MJ, Freeman K (2015). Knowledge gains following a child sexual abuse prevention program among urban students: A cluster-randomized evaluation. American Journal of Public Health.

[CR22] Slade EP, Wissow LS (2007). The influence of childhood maltreatment on adolescents’ academic performance. Economics of Education Review.

[CR23] Tavakol M, Dennick R (2011). Making sense of Cronbach's alpha. International Journal of Medical Education.

[CR24] Topping KJ, Barron IG (2009). School-based child sexual abuse prevention programs: A review of effectiveness. Review of Educational Research.

[CR25] U.S. Department of Health & Human Services, Administration for Children and Families, Administration on Children, Youth and Families, Children’s Bureau. (2019). Child Maltreatment 2017. https://www.acf.hhs.gov/cb/research-data-technology/statistics-research/child-maltreatment

[CR26] Walsh, K., Zwi, K., Woolfenden, S., & Shlonsky, A. (2015). School-based education programmes for the prevention of child sexual abuse. *Cochrane Database of Systematic Reviews.*10.1002/14651858.CD004380.pub3. 10.1002/14651858.CD004380.pub3 PMC9805791 25876919

[CR27] White, C., Shanley, D. C., Zimmer-Gembeck, M. J., Walsh, K., Hawkins, R., Lines, K., & Webb, H. (2018). Promoting young children’s interpersonal safety knowledge, intentions, confidence, and protective behavior skills: Outcomes of a randomized controlled trial. Child Abuse & Neglect, 82, 144–155. 10.1016/j.chiabu.2018.05.024 29902697

[CR28] Wildeman, C., Emanuel, N., Leventhal, J. M., Putnam-Hornstein, E., Waldfogel, J., & Lee, H. (2014). The prevalence of confirmed maltreatment among U.S. children, 2004 to 2011. *JAMA Pediatrics, 168*(8), –706. 10.1001/jamapediatrics.2014.410 PMC5087599 24887073

